# Results of a massive experiment on virtual currency endowments and money demand

**DOI:** 10.1371/journal.pone.0186407

**Published:** 2017-10-18

**Authors:** Nenad Živić, Igor Andjelković, Tolga Özden, Milovan Dekić, Edward Castronova

**Affiliations:** 1 Nordeus LLC, Bulevar Mihajla Pupina 165v, Belgrade, Serbia; 2 University of Niš, Faculty of Science and Mathematics, Višegradska 33, Niš, Serbia; 3 University of Belgrade, School of Electrical Engineering, Bulevar kralja Aleksandra 73, Belgrade, Serbia; 4 University of Amsterdam, Faculty of Economics, Roetersstraat, 1018 Amsterdam, The Netherlands; 5 University of Belgrade, Faculty of Political Science, Jove Ilića 165, Belgrade, Serbia; 6 Indiana University, Media School, 601 E Kirkwood Ave. Bloomington, Indiana, United States of America; Universidad de Castilla-La Mancha, SPAIN

## Abstract

We use a 575,000-subject, 28-day experiment to investigate monetary policy in a virtual setting. The experiment tests the effect of virtual currency endowments on player retention and virtual currency demand. An increase in endowments of a virtual currency should lower the demand for the currency in the short run. However, in the long run, we would expect money demand to rise in response to inflation in the virtual world. We test for this behavior in a virtual field experiment in the football management game Top11. 575,000 players were selected at random and allocated to different “shards” or versions of the world. The shards differed only in terms of the initial money endowment offered to new players. Money demand was observed for 28 days as players used real money to purchase additional virtual currency. The results indicate that player money purchases were significantly higher in the shards where higher endowments were given. This suggests that a positive change in the money supply in a virtual context leads to inflation and increased money demand, and does so much more quickly than in real-world economies. Differences between virtual and real currency behavior will become more interesting as virtual currency becomes a bigger part of the real economy.

## Introduction

In recent years the research value of large-scale online environments has become more apparent. [1–3] Online communities can contain hundreds of thousands (if not millions) of users for long periods of time. The owners of these communities design controlled experiments (“A/B tests”) to test the commercial effects of different design decisions. The same protocol can be used to investigate issues of academic and real-world interest. As virtual currencies such as Bitcoin grow in importance, the behavior of monetary in these virtual systems will become more significance. Policymakers will want to remain alert to the possibility that real-world intuitions about money may, in the virtual setting, be false. [4–6]

In this paper we explore the common intuition (backed by monetary theory) that when money supply increases, the price of money—the interest rate—must fall. This effect is an important cog in the machine that connects monetary policy to economic stimulus. Increasing money supply is believed to lower the cost of money, which makes borrowing and investing easier. But what if an increase in money supply actually raised the cost of money? This would be possible if, for example, an increase in money supply unleashed powerful inflationary forces. In an inflating economy, demand for currency rises. Rising currency demand leads to a rise in the cost of money, and it is possible that the demand increase is so large that the cost of money is higher after the money supply increase than before it. Indeed, it is believed that sustained money supply increases will have such an effect in the “long run.” In a virtual money environment, however, the “long run” may be quite short.

We use a controlled experiment in the football management game *Top Eleven—Be a Football Manager* to investigate this timing of the response of money demand to an endowment of money. *Top Eleven* is a fairly simple monetary economy. Nordeus, one of Europe’s leading game development studios, owns the game and is the only source of money. Money is provided to new players as an endowment. Players can later purchase more game money at a fixed price in terms of real-world currency (with quantity discounts). This arrangement creates a simple but interesting money market, where supply is flat and demand is downward sloping with respect to an actual price instead of an interest rate.

Experiments can be conducted in this simple economy to explore different commercial, academic, and policy issues. Nordeus conducts experiments primarily to determine the response of virtual money purchases to design changes.

## Online communities, big data, and scientific research

The opportunity to conduct large-scale experiments in games like *Top Eleven* has emerged as a result of several converging trends. First is the availability of massive datasets of human behavior (“big data”). Big data is often defined as data generated in real-time that is less structured and has a larger scale than static datasets of the past. Many believe that big data will transform business, government and other aspects of the economy by improving the way we measure, track and describe economic activity. [7] Massively Multiplayer Online Games (MMOGs) and other forms of massively multi-user virtual environments allow the incorporation of big data into economic analysis by investigation of customer behavior and the associated economic modeling on a societal scale. Mostly due to a lack of access to large-scale datasets, previous work on behavioral economics has been limited to building simulations. Lehdonvirta [8] argues that, while agent-based computational simulations have been widely used by economists, modeling human behaviour is a hard challenge and the validity of results from such studies remain limited. Consequently, there has been an increasing number of studies on the analysis of economic behavior using large datasets in online games.

A second trend is the emergence of the free-to-play business model in the game industry, which allows companies to rapidly perform experiments. Traditionally, games were treated as any other product, being sold initially for a fixed, predetermined price, and then consumed for some time period. The free to play model relies on a long term, service-like, relationship between a game and a user. Free to play games offer full and free access to all the important aspects of the game to any user willing to download and play. They generate revenue by “monetizing” the players, that is, inducing them to spend real money on virtual items that enhance the player experience. Sometimes these virtual items have direct gameplay effects (more effective armor); sometimes they are purely cosmetic (better looking armor). Usually companies use a two-currency model: real-world currency is used to buy an in-game virtual currency, which in turn is used to buy virtual items. Two aspects of this situation are appealing from a scientific perspective. First, the fact that these games are downloaded free of charge means that the potential user base is very big, orders of magnitude bigger than the user bases of traditionally sold games. This fact opens up possibilities for large experiments on these user bases. Second, the existence of economies with virtual currencies is possibly a fertile ground for some general economic conclusions if solid theoretical analogies between virtual and real economies can be established.

Third, as large-scale research in games has proceeded, it has indeed established strong parallels between economies in games and in the real world. After industry analysts first began making comparisons between game economies and the real world [9], academics began studying the issue closely. Castronova [10] used market data from online auction sites to show that consumers treat virtual currencies as having real-world economic value. Similarly, Yamaguchi [11] and Nash & Schneyer [12] established parallels between valuation of virtual goods in games and valuation of goods in the real world. Later, Castronova et. al. [5] used data from a large virtual economy to show that macroeconomic behavior in virtual economies closely follows the predictions of standard models of macroeconomic theory. Lehdonvirta [13] showed that consumers are influenced by the features and attributes of virtual items in the same way as they are influenced by the features and attributes of real-world items. For example, while the functions of virtual items affect perceived quality, so do its cosmetic features, in the same way that automobile consumers are influenced both by the speed of a car as well as its looks.

In recent years this literature has expanded in scope. Drachen et. al. [14], document the changes in the in-game economy and showing how players migrate into and out of the economy across the lifetime of a browser-based online game. Levitt et. al. [1] carry out a field experiment on quantity discounts in a game involving over 14 million consumers. The findings support some aspects of received economic theory but also toss up some surprises, such as a lack of response by purchases to large price discounts. An unstated assumption in their work, published in the prestigious *Proceedings of the National Academy of Sciences* is that the economic behavior of game communities is of legitimate academic interest. This represents a considerable paradigm shift from the period 2000-2010, when economic behavior in online games was not considered “real.”

## Theoretical considerations

Our primary theoretical focus is on the question of whether endowments of money increase or decrease the demand for money in a short time frame. Virtual environments provide a unique testbed for monetary policy questions: While the underlying economic drives are the same in real-world and virtual contexts, the structure of the money market is quite different.

**Fig 1:**
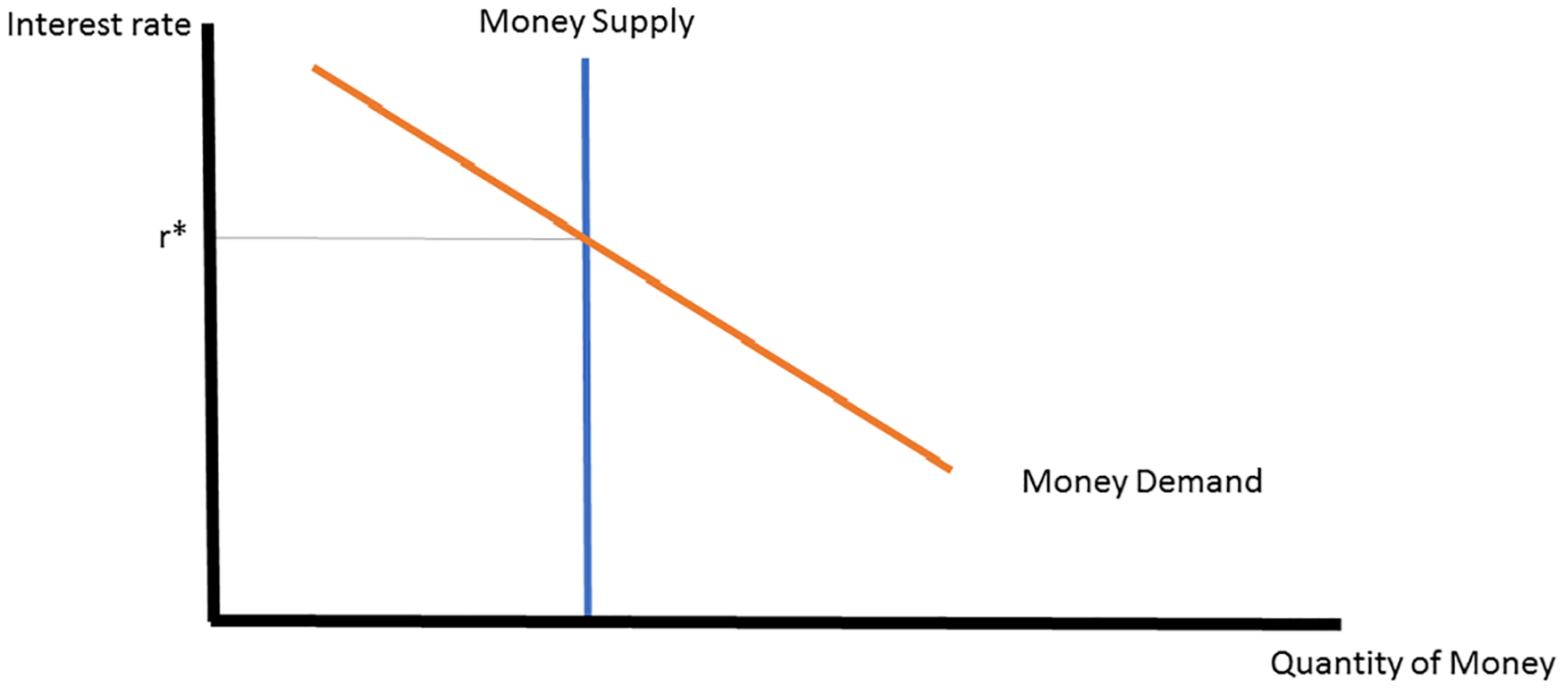
Traditional money market.

Fig 1 shows the traditional money market graph from introductory macroeconomics. Money demand is downward sloping with the interest rate, and money supply is fixed at a given quantity. The intersection of money supply and demand yields the equilibrium interest rate. An increase in money supply unambiguously reduces the interest rate.

**Fig 2:**
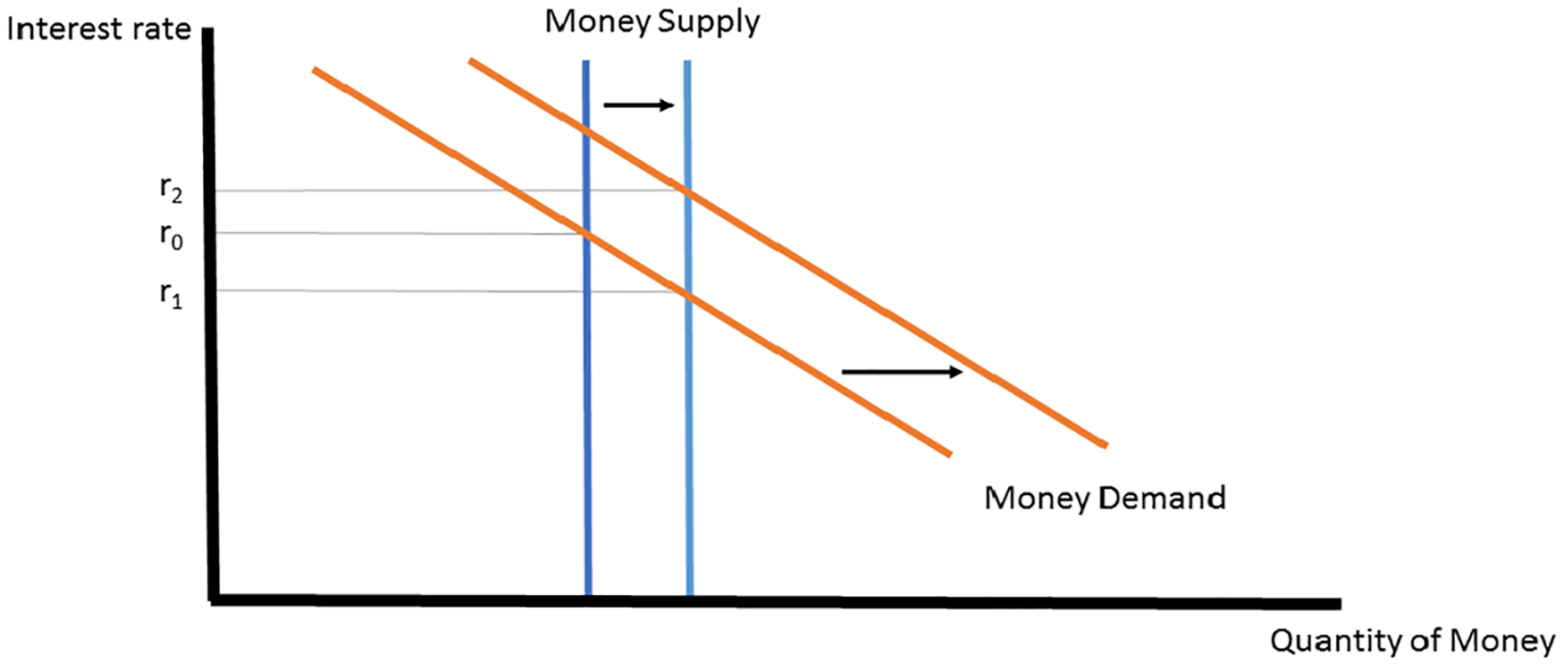
Money supply increase raises interest rates.

This short run model assumes that money demand is stable. However, over time, an increase in money supply may cause an increase in the price level. In this case, money demand will rise. Fig 2 illustrates a case where the increase in money demand is so great that the interest rate actually rises after an increase in money supply and the attendant inflationary effect on money demand.

**Fig 3:**
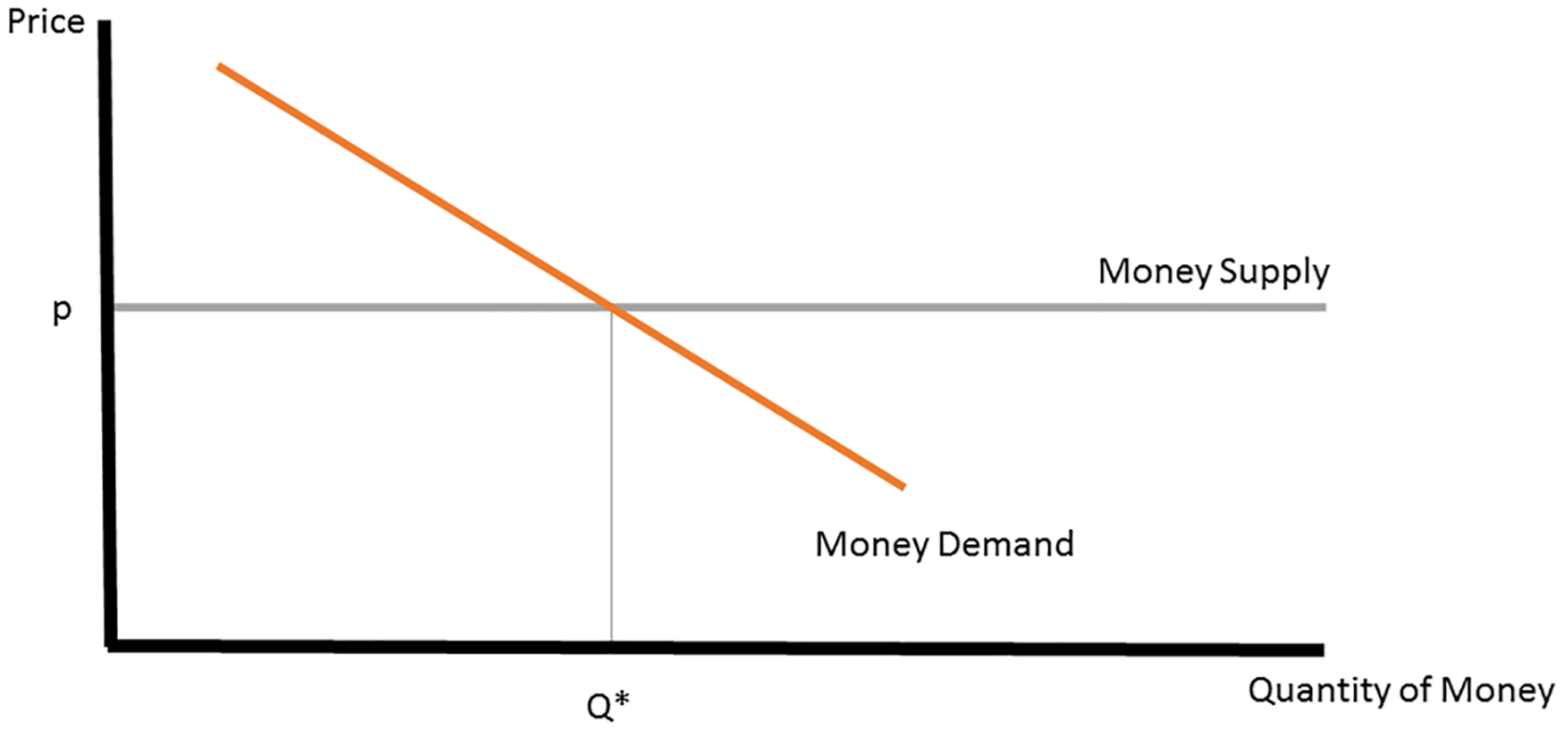
Virtual currency market.

**Fig 4:**
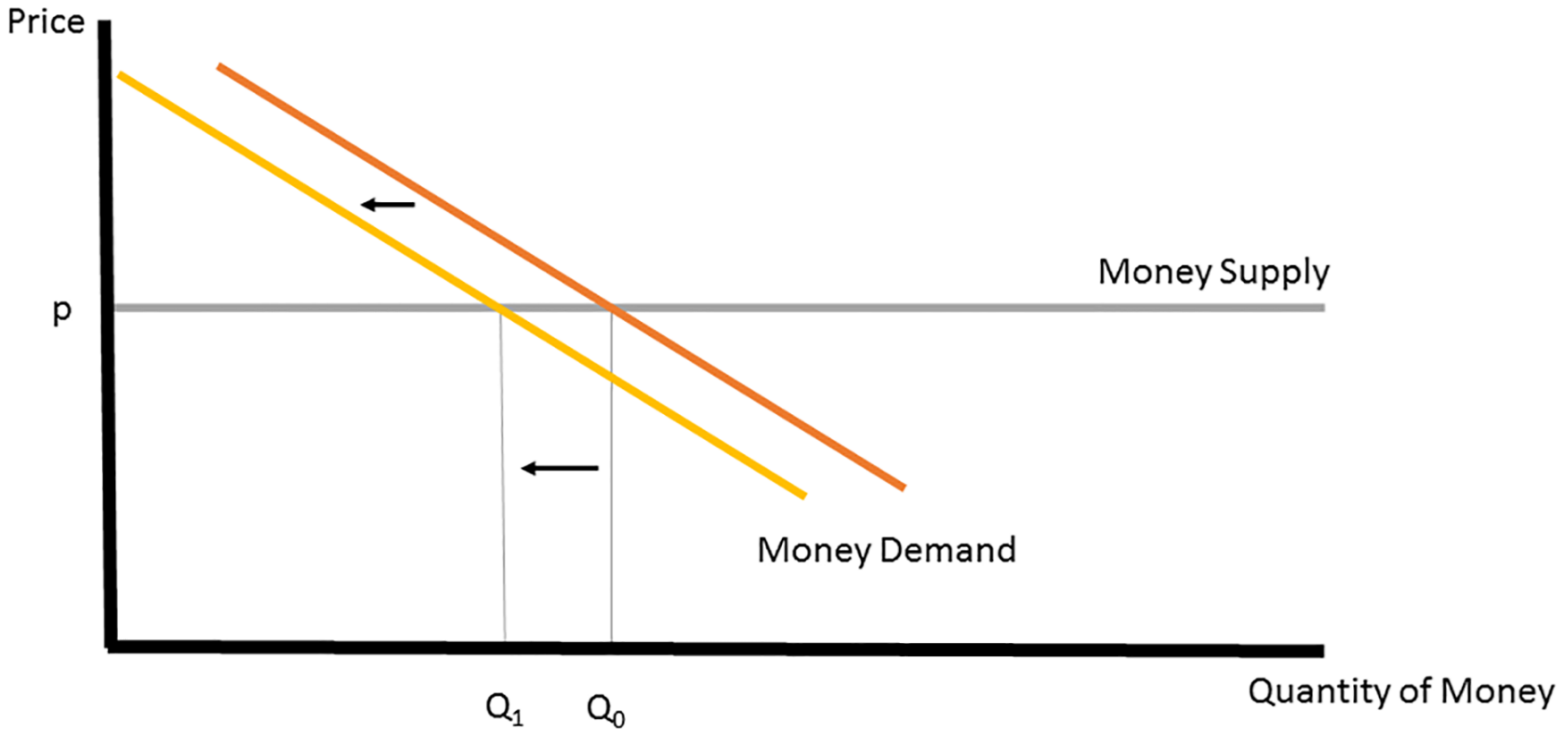
Virtual currency market with money endowment.

Fig 3 shows how these effects appear in virtual money markets, which are distinguished by infinite rather than fixed money supply. In a virtual currency market, consumers satisfy their demand for virtual money by purchasing it using real-world money. Money demand is downward sloping in the price of money, which is fixed by the currency owner. In the virtual context, policymakers have two ways to increase liquidity: Either by lowering the fixed price of virtual money, or by giving virtual money away for free. The former would be modeled by a downward shift in the supply line, leading to more virtual money purchases. The latter effect, of giving money away, would appear as a leftward shift in money demand, as depicted in Fig 4. Giving money away in this fashion unambiguously reduces consumer expenditures on virtual currency. Reduced consumer expenditures imply lower real-money revenues for the game owner. The possibility of lower real-world revenues makes owners of virtual currency skittish about providing large endowments of their currency to new players.

**Fig 5:**
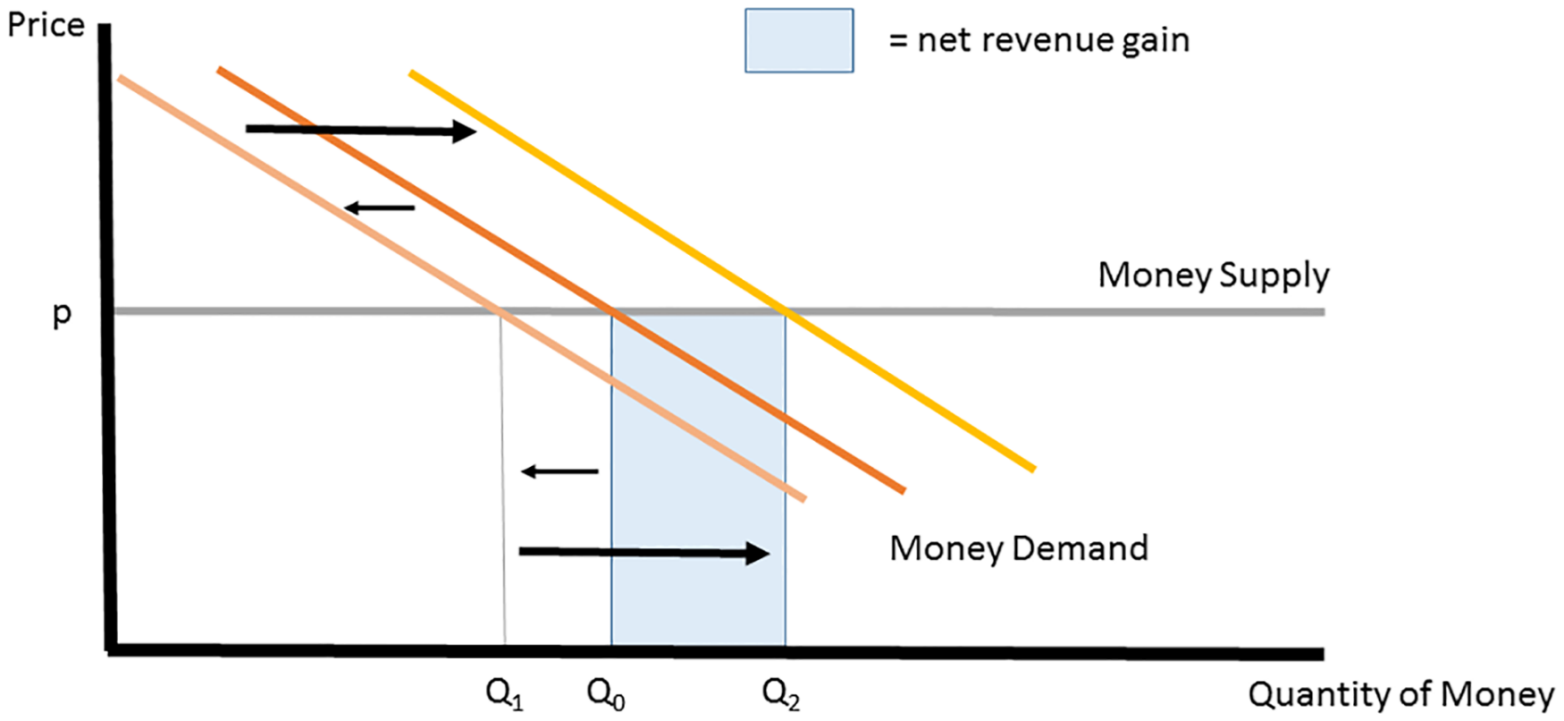
Virtual currency market with money endowment and increased money demand.

In recent years, however, it has come to light that the currency-sales revenues of virtual currency owners can be increased by giving away substantial initial endowments of the currency. Fig 5 shows how this must work: The virtual currency endowment must lead to a net increase in money demand, which suggests a money demand function that increases non-monotonically as the quantity of money is increased. We will now develop a simple model that can explain this possibility.

### A stylized model of virtual money demand

Demand for money in games and other virtual currency environments is driven by the desire for the items can be purchased by virtual money. Money demand would also be driven by player incomes and tastes, but these effect likely do not change substantially in the course of the experiment. In terms of modeling, we take them as fixed.

Our virtual economy is made up of two main parts: The money demand side, which consists of customers that purchase virtual tokens to engage in virtual player transactions; and the money supply side, which consists of the profit-maximizing company. For simplicity, suppose the preferences of the customers can be aggregated by a representative player. The player enjoys having more virtual tokens, since this makes him more competitive on the auction markets and allows him to enjoy the game more. However, he dislikes spending actual money on these tokens. These two ideas can be incorporated by using a standard utility function as follows:

$$\begin{array}{c} U\left(C,L\right)=\frac{C^{\sigma }}{\sigma }-L \end{array}$$ (1)

where *C* and *L* denote the virtual consumption and the actual money spent on the virtual tokens respectively. *σ* is a parameter that governs the marginal utility of consumption: When *σ* < 1, consumption is characterized by decreasing marginal utility, implying each additional unit of consumption brings happiness at a decreasing rate.

Denote by *ω* the initial endowment that the company provides to the player at the beginning of the game. Accordingly, the player is able to make virtual transactions using this endowment, and the tokens he purchases himself. Assuming he spends all of the virtual money available, his budget constraint is characterized by:

$$\begin{array}{c} \omega +L=C \end{array}$$ (2)

Hence the player wants to maximize *U*(*C*, *L*) subject to this budget constraint. Plugging (2) into (1) yields the following maximization problem in actual money spent *L* for the player, for a given level of endowment *ω*:

$$\begin{array}{c} \underset{L}{m}ax\;\;U\left(L\right)=\frac{{\left(w+L\right)}^{\sigma }}{\sigma }-L \end{array}$$ (3)

A straight-forward computation yields the corresponding first-order optimality condition associated with this problem:

$$\begin{array}{c} {\left(w+L\right)}^{\sigma -1}=1 \end{array}$$ (4)

The left-hand side of (4) is the marginal utility of *L*, while the right-hand side denotes the marginal disutility. It is readily seen that a higher initial endowment *ω* unambigiously decreases the marginal utility, while leaving the marginal disutility unchanged. Therefore, the ceteris paribus effect of higher *ω*, i.e. an increased quantity of money, is a lower level of player expenditure. In fact, solving for *L* simply yields the equilibrium level of expenditure L* as a function of *ω*:

$$\begin{array}{c} L^{*}=1-\omega \end{array}$$ (5)

Next we turn to the money supply side of the economy, characterized by the company’s choice of endowment *ω*. Given its profit-maximizing nature, the company’s problem is summarized as:

$$\begin{array}{c} \underset{\omega }{m}ax\;\;L^{*}\left(\omega \right) \end{array}$$ (6)

In other words, the company simply picks *ω* at the level that yields the highest level of consumer expenditure in (5). Since the money demand is decreasing in money supply, the equilibrium strategy for the company trivially reduces to setting ω* = 0, that is, to provide no endowment at all.This corresponds to the case of infinitely elastic money supply of Fig 3.

In the above discussion, we assume the player strictly dislikes spending money on the game at a constant rate. This is the main assumption that drives the result of no endowment as the optimal decision of the company. It is, however, plausible that the player’s disutility is dependent on the endowment itself. This could be due to, for instance, anchoring the preferences on the endowment provided. In such a case, a higher level of *ω* would lower the disutility associated with *L*. To incorporate this idea, we augment the player’s utility maximization problem as follows:

$$\begin{array}{c} \underset{L}{m}ax\;\;U\left(L\right)=\frac{{\left(w+L\right)}^{\sigma }}{\sigma }-w^{\alpha }L \end{array}$$ (7)

where *α* determines the impact of the endowment on the player’s expenditure *L*. When *α* = 0, Eq (7) reduces to the previous case. However, when *α* < 0, a higher initial endowment implies lower disutility associated with spending money.

Another assumption that we implicitly used so far is that the in-game price of virtual items remains constant as the money supply is varied. Some games, however, allow player markets to determine the prices of items. Players bid against one another in auction markets. This is the case in *Top Eleven*, where players, acting as managers, bid against one another to secure the services of virtual footballers. As a result, the price of virtual goods (virtual footballers) is not fixed by the company. In general, in an auction-based system, if the company increases the supply of virtual money (by making it cheaper in terms of real-world money), it will cause the price of virtual items to rise. In this experiment, the company increases the supply of money not by lowering its price across the board, but by providing new players with an endowment of virtual money for free. This will, in turn, decrease the value of each virtual token. When the prices of the virtual tokens are kept fixed in terms of real money, this effectively makes the virtual tokens more expensive. To incorporate this inflationary effect into our model, we assume −1 < *α* < 0, which implies the impact of the initial endowment on the player’s expenditure becomes less effective with each additional unit of *ω*. The first-order optimality condition associated with this new utility function is now characterized by:

$$\begin{array}{c} {\left(w+L\right)}^{\sigma -1}=w^{\alpha } \end{array}$$ (8)

In Eq (8), the initial endowment now affects both the marginal utility and disutility of spending money on the game, which introduces a trade-off for the company. Solving (8) for *L* yields the optimal level of expenditure L*:

$$\begin{array}{c} L^{*}=w^{\frac{\alpha }{\sigma -1}}-w \end{array}$$ (9)

Now the company chooses the value of *ω* that maximizes L* in Eq (9). Provided *α* ≠ *σ* − 1, the first-order optimality condition is given by:

$$\begin{array}{c} w^{\left(\frac{\alpha }{\sigma -1}-1\right)}=\frac{\sigma -1}{\alpha } \end{array}$$ (10)

Finally, solving for *ω* yields the optimal level of endowment ω*:

$$\begin{array}{c} \omega^{*}={\left(\frac{\sigma -1}{\alpha }\right)}^{1-\frac{\alpha }{\sigma -1}} \end{array}$$ (11)

As long as *α* ≠ *σ* − 1, and under the assumptions −1 < *α* < 0, 0 < *σ* < 1, Eq (11) shows that the equilibrium level of endowment ω* will be positive. Hence, in this setup, our experiment could be seen as a test for the significance of the parameter *α*. While this stylized model shows one plausible way in which a higher money supply might induce more player expenditure, there are also several informal reasons why money endowments might increase money purchases, and indeed they do not exclude one another but might all be operating at the same time.

First, the behavior is rooted in the situation of *arbitrary coherence* that was imposed in the minds of new users of *Top Eleven*. When a new user joins the game, he probably has no reference point about what the *appropriate value* of certain microtransactions is. The reference point for new users in *Top Eleven* was established with initial endowments of Tokens. When you look at, let’s say, the price of an in-game virtual item that costs 69 Tokens, its value looks differently whether you look it from the standpoint of the user with 40 or 120 Tokens on the account. One thing about this part of our explanation remains uncertain and should be further researched: Is there any spillover of reference points across games or do players carry their reference points from other games and virtual experiences?

This anchoring behavior would not explain the ongoing behavior of players who continued to convert real money for virtual tokens. After all, after a few microtransactions a user with larger initial endowment possibly remains without them; so why does he proceed with further purchases at higher rate than players with lower initial endowment? One possibility is that this might be the result of the consistency seeking [15], which in the long run turns out as an addictive behavior. According to the consistency seeking hypothesis, people have an internal preference for making choices that are consistent with their previous course of actions. We believe this thesis especially holds in the conditions of reduced uncertainty in Top Eleven: as buying and spending tokens can give you an advantage over players who spend less, the price of strengthening the belief that spending is justified gradually drops lower.

Finally, a third mechanism possibly at work would be that the perpetuation of spending behavior gradually develops into a “rational addiction”. [16] One of the prerequisites of the rational addiction is that the past consumption experiences increase the desire for the following consumptions. The repetition of pleasant experiences turns the actual behavior to a habit. More important in our case is that the rational addiction model pictures addictive behavior as a rational medication for certain psychological states. Support for this idea comes from unpublished data from internal studies of the Nordeus player base. These data indicate that of the “Big Five” personality traits (openness to experience, conscientiousness, extraversion, agreeableness, and neuroticism), neuroticism is the best predictor of buying and spending tokens.

## Experimental setting

**Fig 6:**
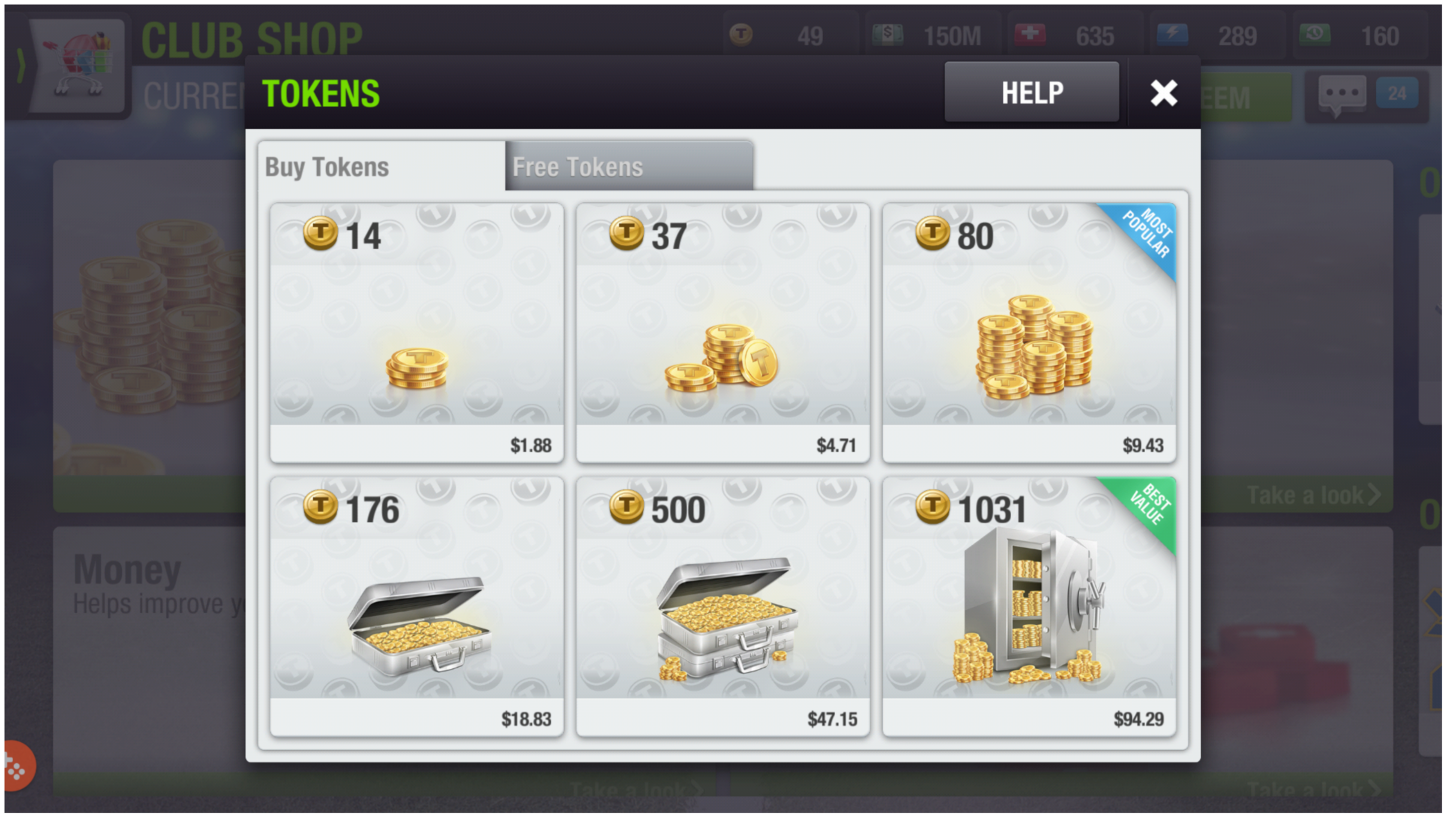
Token packages in Top Eleven.

In order to test for the possibility that an increase in the money supply might increase money demand (and real-world revenues of game companies), We report on an experiment in the game *Top Eleven*. The game is produced by Nordeus, one of Europe’s leading game development companies. It was released in 2010 and has been played by over 150 million players worldwide. In *Top Eleven*, the players are managers of virtual football teams. As a result, *Top Eleven* is a game about managing assets (in this case, virtual footballers) and thus has strategic and economic thinking as its core gameplay. This makes it an interesting setting to observe economic behavior. Much of the mastery in *Top Eleven* lies in intelligently managing and optimally spending virtual assets, of which there are five in total (Aside from tokens, there are four more currencies—rests, used to rest players after matches or training sessions; morale boosters, used to raise morale; treatments, which recover players from injuries; and in-game money, needed to sign player contracts.). We are particularly interested in Tokens, which are used to bid on high quality virtual footballers, as well as purchase player recovery packs and vanity items such as official jerseys and emblems of real clubs. *Top Eleven* offers six Token packages, ranging from around 2$ to around 100$, as seen in Fig 6.

Nordeus regularly designs and releases new virtual footballers, and the virtual managers (the players of *Top Eleven*) engage in competitive bidding to obtain them for their teams. Footballer auctions create demand for the virtual currency, which then drives managers to use real-world money to purchase it. Whenever a manager buys more of these Tokens, he “monetizes,” which is to say, he provides the real-world revenue that Nordeus uses to provide the game. This structure allows Nordeus to rapidly conduct experiments, simply by changing the computer code that runs the game. The experiments produce results at any time scale Nordeus could wish; after a change, the company can observe user behavior five minutes later, or five months, or five years. In addition, the size of *Top Eleven’s* user base allows for experiments on a truly grand scale, with hundreds of thousands of subjects.

Providers of free-to-play online games are primarily concerned with design decisions that maximize revenue. In the present experiment, the design decision in question involves the size of the currency endowment. The endowment is an amount of virtual currency given to each new player upon registration. The question is, how does the endowment affect revenue (which is to say, demand for the virtual currency)? In data terms, revenue is driven by two things. One is participation—the more people participate in a service, generally speaking, the higher the revenues. Participation in the game is measured by **retention**, the degree to which players come back to the game after first joining it. However, revenues are modulated in significant ways by expenditure decisions of the participants. Users must decide to **convert**, that is, to make a real-money purchase; and then they must decide how much real money to spend. Veterans of product-as-service businesses such as online free-to-play games have developed intuitions about the relationship between initial endowments of virtual currency and conversion and retention. Generally, endowments should increase retention up to a saturation point. Giving people more virtual money at the start should encourage them to remain, up to a point. Intuition suggests, however, that endowments should decrease conversion—why would people buy more if they are given more? This seems to create a dilemma in practical terms: Increasing the endowment would seem likely to keep more players in the game, but cause them to spend less. However, as we have argued above, there are formal and informal theoretical reasons why an increase in endowment might not only increase retention but also conversion.

We translate these general concepts of retention and conversion into specific metrics as follows.

- **Day 1 Retention**: The percentage of new users who log in to the game one day after their initial registration.
- **Day 7 Retention**: The percentage of new users who log in to the game on the seventh day after their initial registration.
- **Daily Conversion**: The percentage of active users who make their first real-money purchase on a given day.
- **Conversion Magnitude**: The average size of the conversions that occur on a given day.

Some examples will clarify what is meant by these quantities. Suppose a game has 100 players and on Day 0, 10 new players sign up. We track those new players and determine that, on Day 1, 5 of them logged into the game at least once. This means that Day 1 Retention is 0.50. On Day 7, only 3 of the ten new players logged in, making the Day 7 Retention 0.30. Note that the players who logged in on Day 7 may be completely different from those who logged in on Day 1. Moreover, these players may have been in and out of the game over the course of the week, since these measurements do not keep track of continuous participation. Retention simply measures what fraction of new players appear in the game on a given later date.

Now suppose that, of our 10 new players, 2 of them make their first real-money purchase on Day 1. In addition, 13 other players, who have already been in the game, also make their very first purchase on Day 1. This means that 15 players have converted on Day 1, making the daily conversion for that day 0.15. Finally, let the average real-money amount spent by these 15 players be 20$. In that case, the Conversion magnitude for Day 1 is 20$.

In terms of the endowment change, we provide data from a control case as well as two tests. *Top Eleven* has been granting 40 Tokens at the beginning from late 2014 until the experiment discussed in this paper took place. The A/B test was designed to have three groups. The first one is the Control group, with default settings, where every player got 40 Tokens upon registration. For simplicity, this group will be referred to as **Control 40** later in the text. The second and third groups were given 80 and 120 Tokens upon registration, and are referred to as **Test 80** and **Test 120**. The test lasted for 28 days, which is exactly one *Top Eleven* season (this is equivalent to one year season in real life football). Each group had the same number of registered users, and all the other conditions besides initial amount of currency were the same. During the 28 days of testing, approximately 575,000 players were assigned to one of the three groups (In this research protocol, no personally identifiable data are collected. As a result, prior consent was not obtained. This protocol has been reviewed by the Internal Review Board of Indiana University, and has received exempt status.).

## Results and discussion

Table 1 summarizes the results of the experiment. Generally speaking, the increases in endowment raised retention and conversion relative to the 40-Token control group.

**Table 1:** Overall results of the experiment. All real values are normalized to Control 40 group.

Group	D1 Retention	D7 Retention	Daily Conversion	Conversion Magnitude
Control 40	100.0	100.0	100.0	100.0
Test 80	104.5	106.9	108.0	113.9
Test 120	105.3	107.7	104.3	134.8

Focusing first on retention, D1 Retention was raised 4.5 percent by the 80-Token endowment and 5.3 percent by the D7 Retention was even higher, increasing by 6.9 percent for the 80-Token endowment and 7.7 percent for the 120-Token endowment. These are large and substantively significant increases: Reichheld & Schefter [17] indicate that increasing retention rates by only 5 percent increases profits by 25 to 95 percent on average, depending on the business sector. This implies the changes resulting from our experiment for both test 80 and test 120 groups are economically significant in terms of industry averages. More than anything else, these results suggest that the 40-Token endowment was far less than optimal in terms of retention. The data confirm the suspicion that virtual money endowments increase retention up to some point of saturation; a 40-Token increase from 40 to 80 has a much larger effect than a similar 40-Token increase from 80 to 120. This suggests that the optimal endowment in terms of retention probably lies somewhere in the 80-120 Token range.

The data also suggest that the lower endowment was less than optimal in terms of conversion. Daily conversion percentages were 8 percent larger in the 80-Token condition, though only 4.3 percent larger in the 120-Token condition. According to a recent report by the marketing research company *Marketing Sherpa*, the average conversion rate is 2 to 8 percent for e-commerce businesses. [18] This implies that our A/B tests had the impact of a successful marketing campaign in terms of conversion rates. However, the largest substantive effect in the experiment is on conversion magnitudes. New players who made a first purchase in *Top Eleven* during this experiment increased the size of that purchase by 14 percent in the 80-Token condition and 34.8 percent in the 120-Token condition. These conversion effects suggest that the endowment increase had a very large positive impact on the demand for Tokens.

Table 2 compares effects under the different conditions in terms of statistical significance. The findings in terms of retention indicate that the two experimental conditions are statistically significantly different from the control condition, but not from each other. The daily conversion effects are not statistically significant in any condition/control pairing. However, the conversion magnitude is statistically significant in the comparison of the 120-Token condition and the 40-Token control.

**Table 2:** Results summarized by statistical significance.

Metric	Group A	Group B	Outcome	p-value
**D1 Retention**	**Test 80**	**Control 40**	**greater by 4.5%**	**0.005**
**D1 Retention**	**Test 120**	**Control 40**	**greater by 5.3%**	**0.006**
D1 Retention	Test 120	Test 80	greater by 0.7%	0.482
**D7 Retention**	**Test 80**	**Control 40**	**greater by 6.9%**	**0.015**
**D7 Retention**	**Test 120**	**Control 40**	**greater by 7.7%**	**0.005**
D7 Retention	Test 120	Test 80	greater by 0.7%	0.403
Daily Conversion	Test 80	Control 40	greater by 8.1%	0.054
Daily Conversion	Test 120	Control 40	greater by 4.0%	0.197
Daily Conversion	Test 120	Test 80	smaller by 3.8%	0.193
Conversion Magnitude	Test 80	Control 40	greater by 14.0%	0.057
**Conversion Magnitude**	**Test 120**	**Control 40**	**greater by 34.8%**	**0.005**
Conversion Magnitude	Test 120	Test 80	greater by 18.3%	0.059

The practical significance of the retention findings can be seen by simulating how a game company’s revenues would be affected by changes of this magnitude. We will take the Test 120 group as an example. Suppose that, after our experiment, Nordeus decided to give 120 Tokens to new users when they start playing Top Eleven. Our results suggest that day-7 retention of these players will increase by 7.7 percent.

The total revenues of a game is equal to the number of players times the average player’s “lifetime value” or LTV. This is the present value of the future cash flows attributed to the customer during his/her entire relationship with the company. [19] In a simple, short-run context, the LTV is the average daily spending of a player times the number of days played. Therefore, increasing day-7 retention by 7.7 percent implies a 7.7 percent increase in average days played, which implies a similar percentage increase in LTV.

Now imagine a small game with 100,000 players a month, where the LTV is $10. [20] A 7.7 percent increase in LTV yields $10.77 of revenue per player. This translates to $77,000 per month, or $924,000 per year in increased revenue. These revenues accrue almost entirely as profit, because the marginal cost of adding players in these systems is virtually zero. In sum, our hypothetical company can earn almost $1 million in annual profit simply by giving new players 120 Tokens rather than 40.

## Conclusion

A massive online game experiment has provided statistically significant evidence that increasing the endowment of virtual currency increases participation in the game as well as purchases of the virtual currency itself. The former effect is not necessarily surprising, as an increase in purchasing power within a game makes the game more attractive to players. The second effect, that an endowment of money could increase purchases of money, is more surprising. However, it can be understood as the result of a positive shift in money demand, caused by several economic and psychological mechanisms. From this point, it is hard to precisely determine which of the factors played a fundamental role in shaping the behavior we presented. It makes much more sense to think of the behavior as an aggregated outcome of several interacting psychological mechanisms.

However, there are additional questions that remain unanswered and that deserve further research. First, is there any spillover effect of the initial price anchors across various virtual worlds or how previous experiences of price anchors determine the perception of new values in a new virtual world? How should previous experiences look like when you want to nudge people towards seeking the consistency with their previous decisions? Are there any cognitive barriers in people’s minds that might prevent them to spend as we might want to nudge them? [21] What are further institutional and economic conditions that must be met if we want to see people behaving the way we expect from them and in a way that we observed in our study. These questions seek further clarifications.

Our experimental setup can be easily extended to research other issues. One issue, important from a business as well as academic point-of-view, is the impact of *demographic heterogeneity* across customers. It would be interesting to know how different players respond to changes in virtual money endowment. This would shed more light on the optimal pricing strategies that should be adopted revenues. Another promising point is the impact of changing money supply on the *transitional path* of retention rates from the short-run to the long-run. While our current results indicate that money supply has an impact on the retention rates both in the short- and long-run across all three test groups, it could very well be the case that the transitional paths exhibit different characteristics across all test groups. Finally, while we are able conclude that the maximum retention potential saturates on the interval [80, 120], we are not able to pinpoint the exact location of this optimal point. Therefore introducing new test groups across this interval could lead to even more fruitful results in future research.
